# A fully convolutional network for weed mapping of unmanned aerial vehicle (UAV) imagery

**DOI:** 10.1371/journal.pone.0196302

**Published:** 2018-04-26

**Authors:** Huasheng Huang, Jizhong Deng, Yubin Lan, Aqing Yang, Xiaoling Deng, Lei Zhang

**Affiliations:** 1 College of Engineering, South China Agricultural University, Guangzhou, China; 2 National Center for International Collaboration Research on Precision Agricultural Aviation Pesticide Spraying Technology, Guangzhou, China; 3 College of Electronical Engineering, South China Agricultural University, Guangzhou, China; 4 College of Agriculture, South China Agricultural University, Guangzhou, China; Instituto Agricultura Sostenible, SPAIN

## Abstract

Appropriate Site Specific Weed Management (SSWM) is crucial to ensure the crop yields. Within SSWM of large-scale area, remote sensing is a key technology to provide accurate weed distribution information. Compared with satellite and piloted aircraft remote sensing, unmanned aerial vehicle (UAV) is capable of capturing high spatial resolution imagery, which will provide more detailed information for weed mapping. The objective of this paper is to generate an accurate weed cover map based on UAV imagery. The UAV RGB imagery was collected in 2017 October over the rice field located in South China. The Fully Convolutional Network (FCN) method was proposed for weed mapping of the collected imagery. Transfer learning was used to improve generalization capability, and skip architecture was applied to increase the prediction accuracy. After that, the performance of FCN architecture was compared with Patch_based CNN algorithm and Pixel_based CNN method. Experimental results showed that our FCN method outperformed others, both in terms of accuracy and efficiency. The overall accuracy of the FCN approach was up to 0.935 and the accuracy for weed recognition was 0.883, which means that this algorithm is capable of generating accurate weed cover maps for the evaluated UAV imagery.

## Introduction

Many agricultural crops require the use of herbicides as essential tools for maintaining the quality and quantity of crop production [1]. However, the inappropriate use of herbicides could cause yield reduction and environmental pollution. The main reason for this problem is that, the usual practice of weed management is to broadcast herbicides over the entire field, even within the weed-free areas [2]. In the field of Site Specific Weed Management (SSWM), there is a need for developing a new strategy to solve the problem of current weed control practices.

To achieve this goal, it is necessary to generate an accurate weed cover map for precise spraying of herbicide. In the practice of SSWM, a weed cover map can be used to decide where the chemicals are needed most, least, or where it should not be used at all [3]. Usually, the weed cover map can be developed using remote sensing technology. Through image processing, remote sensing imagery can be converted to a weed cover map [3] which could be applied for accurate spraying.

In the past few years, piloted aircraft and satellite remote sensing have been employed for weed detection and mapping [4, 5]. However, it is hard to obtain a satisfactory result because of the insufficient spatial resolution of the remote sensing imagery. Today, the insufficiency of spatial resolution can be appropriately solved by using UAV-based remote sensing technology. UAVs can fly at a low altitude and capture ultra-high spatial resolution imagery, which may significantly improve the overall accuracy of weed mapping. Pérez-Ortiz et al. [6] utilized the UAV imagery for weed mapping, and an excellent performance was obtained using a semi-supervised approach. Castaldi et al. [7] applied UAV multispectral imagery for maize/weed classification. The classification results used in the weed management of the maize field leaded to a decrease in the use of herbicide without negative consequences in terms of crop yield.

Weed detection in crop fields is a difficult task. This task is even complex in rice fields due to that the rice and weeds share similarities in spectral characteristics and general appearance, and due to the variability and changing conditions in rice fields. Even using OBIA method, accurate weed mapping is still a challenging task. In recent few years, machine learning methods have been successfully applied in weed mapping based of UAV imagery. Alexandridis et al. [8] applied four novelty detection classifiers for weed detection and mapping of Silybum marianum (S. marianum) weed based on UAV multispectral imagery, and the identification accuracy using One Class Support Vector Machine (OC-SVM) reached an overall accuracy of 96%. Tamouridou et al. [9] used the Multilayer Perceptron with Automatic Relevance Determination (MLP-ARD) to identify the S. marianum among other vegetation based on UAV remote sensing, and the S. marianum identification rate was up to 99.54%.

As the varieties of machine learning approaches, Convolutional Neural Network (CNN) is now the dominating method for most remote sensing applications [10–12]. However, traditional CNN architecture is an “image-label” mode that maps an image into a 1-D probability distribution related to different classes [13], while what we expect in our application is a dense class map, which is a “pixel-label” pattern. In order to generate a weed cover map, the output image should have the same resolution with the input image, and the output map is a 2-D probability distribution. To perform the “pixel-label” classification task, Shelhamer et al.[14] proposed a Fully Convolutional Network (FCN) which is capable of providing dense class map in image analysis. Gang Fu et al.[13] applied FCN method for classification of satellite imagery and proved that this approach outperformed other existing methods in terms of accuracy. Sherrah et al.[15] used an adapted FCN without downsampling for semantic labeling of aerial imagery and proved that the proposed network yielded state-of-the-art accuracy for the ISPRS Vaihingen and Potsdam benchmark data sets. However, to the best of our knowledge, the potential of FCN method for weed mapping of UAV imagery is not yet been accessed.

The objectives of this paper are to: (1) Evaluate the feasibility of FCN method, which is usually used in computer vision field, on application of accurate weed mapping from the UAV imagery; (2) Apply transfer learning method to improve the generalization capability; (3) Use skip architecture to increase the prediction accuracy; (4) Compare the proposed method with other methods in the application of weed mapping.

The rest of this paper is organized as followed: Section 2 introduces the data collection and analyzing methods; Section 3 describes and discusses the experimental results; and Section 4 draws the conclusion and future perspective.

## Materials and methods

### Study site

**Fig 1:**
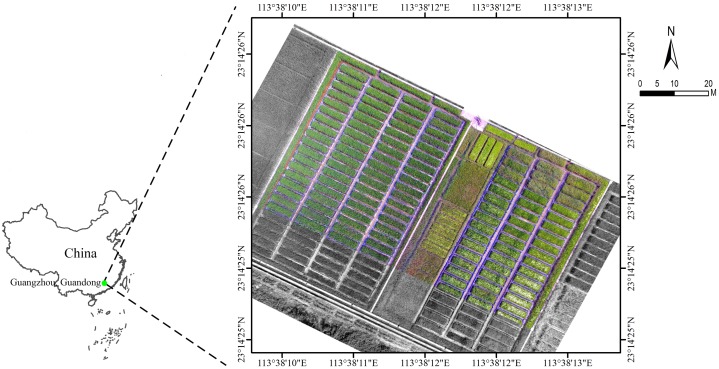
The general location of the study site and the brief overview of the studied rice field.

**Fig 2:**
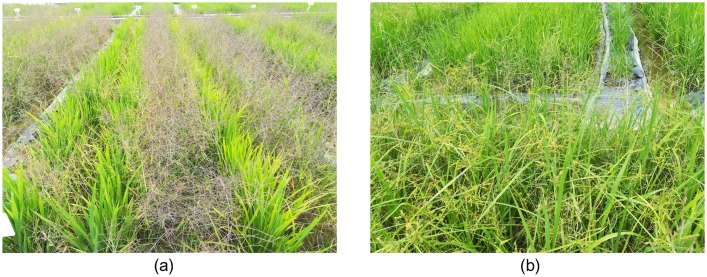
In-field photograph of the study site. (a) The cultivated rice and some patches of L. chinensis; (b) The cultivated rice and some patches of Cyperus iric.

The study site was located at Zengcheng Teaching and Research Bases, South China Agricultural University (Guangzhou city, Guangdong province, China, coordinates 23.240 N, 113.637 E, datum WGS84). The UAV data from a rice field was studied. This plot had an area of 0.54 ha, and was divided into 172 subplots of approximately the same size of 20 m^2^ (Fig 1). These subplots were all irrigated and sown (Huahang No. 31 [16]) on August 21^th^ 2017, at 60 Kg ha^-1^ in rows spaced 50 cm apart. The fertilizer (50 Kg N ha^-1^, 50 Kg P_2_O_5_ ha^-1^ and 40 Kg K_2_0 ha^-1^) was drilled at the seedling stage, and N at 40 Kg ha^-1^ was top-dressed at the active tillering stage. The studied field was naturally infested with *Leptochloa chinensis Nees (L*. *chinensis)* [17] and *Cyperus iric* [18] (Fig 2), and the weed species were mixed in the field.

### Data collection

**Fig 3:**
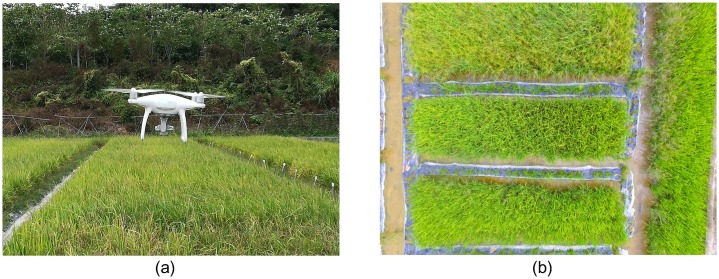
An overview of UAV and the collected imagery. (a) The Phantom 4 UAV flying over the rice field; (b) Aerial imagery (with RGB channels) captured by the UAV at an altitude of 6 m, showing the cultivated rice and some patches of weeds.

A multi-rotor UAV (Phantom 4, SZ DJI Technology Co., Ltd., Shenzhen, China) was used to collect all of the imagery used in this study. The Phantom 4 camera uses 1-inch CMOS sensor to capture 12 megapixel imagery [19] with the resolution of 4000*3000 pixels. During the flight, a 3-axis gimbal on Phantom 4 provided a steady platform to keep the attached camera pointed close to nadir. An overview of the UAV and the captured imagery were shown in Fig 3. DJI GS PRO (also known as the ground station of UAV) is an Ipad app designed for DJI aircrafts, and was used to plan and control automatic flight [20] of UAV in this study.

**Fig 4:**
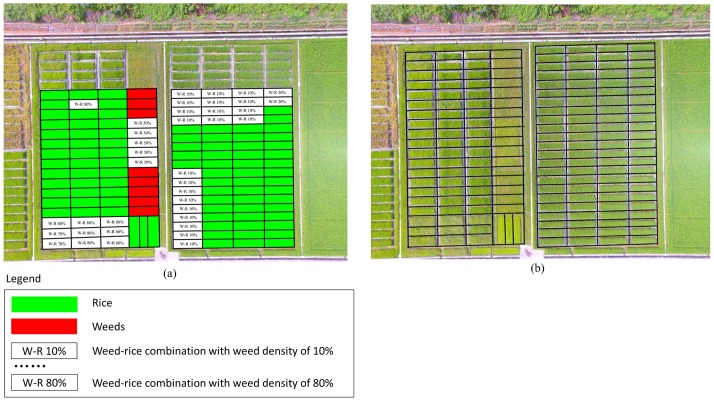
An overview of the results from ground investigation. (a) The ground truth map of the experimental site; (b) Sampling zone for division of the subplots in the rice field.

Field campaigns were conducted on October 2^th^ 2017, just when the rice and the infested weeds were both in growing stage and the herbicide treatments were recommended. The flights were conducted with the permission from Prof. Guohua Zhong, who was the authority responsible for the experiment site. An rectangle region of 60 ╳ 50 m was selected as the experimental area to perform the flights. According to the results of ground investigation, the selected region was composed of 90 subplots (1800 m^2^) of rice, 8 subplots (160 m^2^) of weeds and 38 subplots (760 m^2^) of weed-rice combination with different weed densities, as shown in Fig 4. During the ground investigation, the weed density was obtained by visual judges under the instruction of agronomy experts. It can be seen from Fig 4 that, the experimental site contained much variability in weed-rice combinations.

The coordinate of each corner was collected as a boundary point and the flight plan was automatically planed by DJI GS PRO. During data collection, the flight altitude was kept 6 meters above the ground, and the spatial resolution was around 0.3 cm. Meanwhile, the forward-lap and side-lap were set to 60% and 50% respectively. In this work, 91 imagery were collected in total.

Since the resolution of our imagery is 4000 ╳ 3000 and much larger than other FCN experiments like [14, 15], directly exporting the imagery into our FCN network can easily exhaust the GPU’s memory. Therefore, each original imagery was divided into 12 tiles of size 1000 ╳ 1000, and 1092 tiles were obtained in total. Among the whole imagery tiles, 892 titles were randomly selected as training dataset, and the other 200 tiles were chosen as validation dataset.

**Fig 5:**
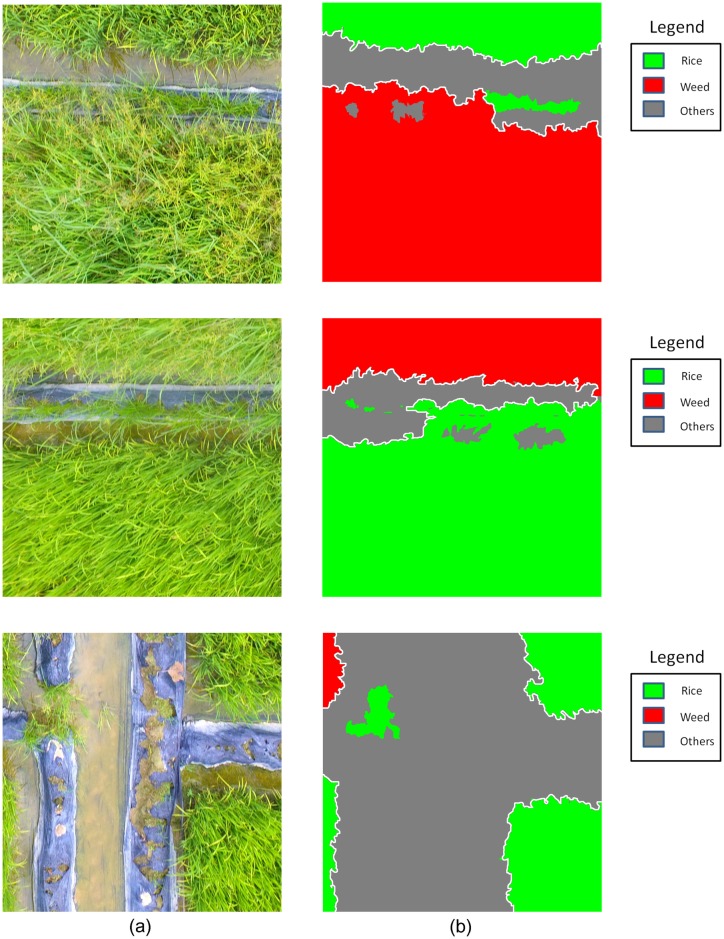
Demonstration for imagery labeling. (a) UAV imagery; (b) GT labels corresponding to the imagery in (a).

In the UAV imagery, all the objects were divided into 3 classes: *Rice*, *Weeds* and *Others* (including cement ground, water, et al.). Since the resolution of imagery is high enough to visually distinguish different classes from the imagery, we manually label the Ground Truth (GT) labels using specified colors: color green for *Rice* class, color red for *Weeds* class, and color gray for *Others* class, as shown in Fig 5(b).

### Methods

**Fig 6:**
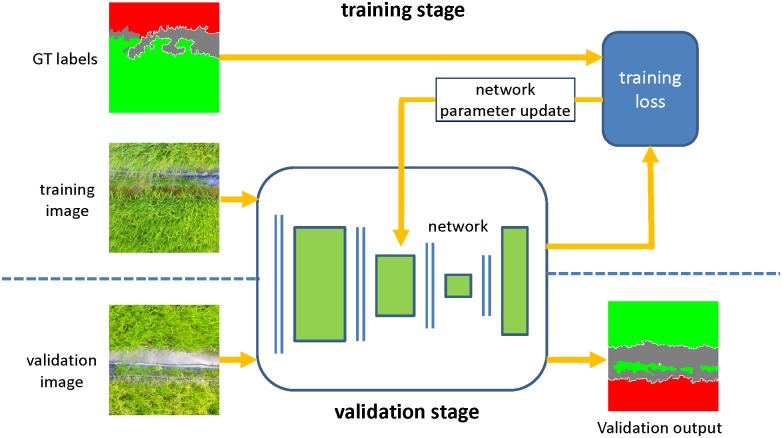
Overview of our methodology: The training stage and validation stage are illustrated in the upper and lower parts, respectively.

Similar with other supervised machine learning approaches, our method could be divided into training and validation stage, as Fig 6. In the training stage, the image-labels pairs in the training set, with pixel-to-pixel corresponds, are input into the FCN network, as is shown in the upper part of Fig 6. The network maps the input image into a same sized output image, and the output image as well as the ground truth label (GT label) are used to compute the loss as an objective function. After that, the gradient of the objective function with respect to the weights of all the network modules are computed using the chain rule for derivatives, and then the network parameters are updated by gradient descend method. The above training process will be iterated until the loss is less than a threshold or the maximum iterations reach. In the validation stage, the trained FCN network will map the validation image into a prediction class map with per-pixel classification, as is shown in lower part of Fig 6.

The FCN network was proposed as the basic architecture for our dense prediction task. Based on the basic of FCN network, transfer learning is introduced to improve the generalization capability, and skip architecture is applied to increase the prediction accuracy.

#### Fully convolutional network

**Fig 7:**
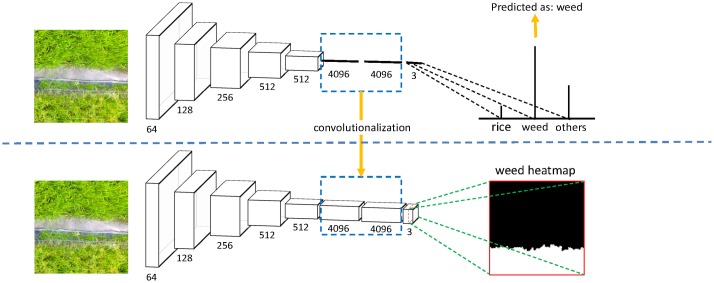
Overview of architecture of CNN (upper part) and FCN (lower part).

The general structure of a typical CNN is composed of multiple convolutional layers interlaced with pooling layers [21], followed by some fully-connected layers in the end [22], as shown in the upper part of Fig 7. The convolutional layer automatically extract the hierarchical and high-level feature representation [21], while pooling layer reduces the image resolution and help achieve spatial invariance, and the fully-connected layer reduces the image dimension and outputs a 1-D distribution over classes. In general, the maximum output in the 1-D distribution corresponds to the predicted result, which classifies one image to one single label. This “image-label” mode has achieved success in the scene classification of high-resolution satellite imagery by the study of Hu et al [11].

However, the goal of this work is not a single label of the input image, but a dense prediction map, which could generate a weed cover map for our UAV imagery. Therefore, the FCN method was proposed for our study. Compared with CNN, FCN network transforms all the fully-connected layers into convolutional layers and enables a classification net to output a heatmap [14]. Using this transformation, the 2-D spatial information of the original image is properly maintained, which contributes to output a dense prediction map. In the last convolutional layer, the number of feature maps corresponds to the quantity of classes, where each feature map represents the spatial probabilities with regard to a certain class, as shown in the lower part of Fig 7.

**Fig 8:**
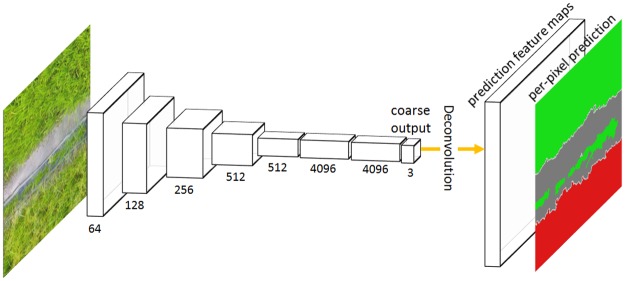
Overview of deconvolutional operation for per-pixel classification tasks.

Though FCN network could maintain the 2-D spatial information, the size of output is typically reduced using the pooling operation. To compensate the resolution reduction, the deconvolution operation is applied to the coarse output, as introduced by Shelhamer et al.[14]. A deconvolutional layer is initialized using bilinear interpolation and learned for nonlinear regression during the training stage. As the upsampling approach, the deconvolutional layers restore the output to full resolution, thus an end-to-end, pixel-to-pixel prediction architecture was established, as shown in Fig 8. The channels of the last deconvolutional layer is equal to the quantity of classes, and each feature map represents the heat map for a certain class (the 3 feature maps corresponds to *Rice*, *Weeds* and *Others*, respectively). For every pixel in the prediction feature maps, the class with maximum probability along the whole channels is used as the predicted labels [15], and the predicted labels of all the pixels in the image form the output result.

During the training process, the entropy-loss was calculated by comparing the target label vectors *y* and predicted label vectors $\hat{y}$ (assuming there are *n* samples and *T* possible classes), as defined by (1):

$$\text{L}=-\frac{1}{n}\sum_{i=1}^{n}\sum_{k=1}^{T}y_{\;k}^{\left(i\right)}log{\hat{y}}_{k}^{\left(i\right)}$$ (1)

After the loss calculation, the gradient of the loss with respect to the weights of all modules in the network was computed using chain rules [22], and used for network parameter updating, as define by (2) and (3):

$${\text{W}}^{\left(n+1\right)}=W^{\left(n\right)}-\Delta W^{\left(n+1\right)}$$ (2)

$$\Delta W^{\left(n+1\right)}=\eta \cdot (d_{w}\cdot W^{\left(n\right)}+d\frac{L(W)}{W^{\left(n\right)}})+m\cdot \Delta W^{\left(n\right)}$$ (3)

Where *W*^(*n*)^ and *W*^(*n*+1)^ denoted the previous weights and current weights, Δ*W*^(*n*)^ and Δ*W*^(*n*+1)^ were the previous increment and current increment, *η* was the learning rate, and *d*_*w*_ and *m* denoted the weight decay and momentum, respectively.

#### Transfer learning

As a fully-supervised method, FCN achieves great success in computer vision field as well as remote sensing. However, with limited training data, fully-supervised training will generally cause the problem of overfitting [23], resulting in pool generalization capability.

Transfer learning uses the existing weights from the model that is fully-trained in a specific dataset like ImageNet [24], and retrains it for new classes [25]. Previous studies have proven that transfer learning is effective in many remote sensing applications [11, 13, 26]. Generally, transfer learning method discards the last layer of the pre-trained model, and appends a fully-connected layer where the the neurons corresponds to the number of predicted classes. During the training stage, final layer is trained from scratch, while the others are initialized from the pre-trained model and updated by back-propagation rule or kept fixed.

In this work, the AlexNet [27] that won the ILSVRC-2012 champion was considered, as well as the VGGnet [28] and GoogLeNet [29] that showed great performance in the ILSVRC-2014. Despite its success, CNNs were largely forsaken by the machine learning communities until the emergence of AlexNet in 2012 [22]. When applied to the ImageNet in ILSVRC-2012, AlexNet halved the error rate of the state-of-art. The success came from the efficient use of GPU, the technique of Local Response Normalization, overlapping pooling, data augmentation and dropout strategy [27]. VGGnet was developed by increasing the depth to 16–19 and using very small (3**╳**3) convolution filters [28], which showed a significant improvement on the classification accuracy and generalization capability. GoogLeNet was a 22 layers deep network that won the runner-up of ILSVRC-2014 classification and detection challenges. The hallmark of this architecture was the improved utilization of the computing resources inside the network, which allowed for increasing the depth and width while keeping the computation costs constant [29]. Compared with AlexNet and VGGnet, GoogLeNet achieved a similar accuracy in ILSVRC with less parameters. In the following experiments, the pre-trained AlexNet, VGGnet and GoogLeNet were adapted into FCN network for the weed mapping task by fine-tuning.

#### Skip architecture

The pooling layers in the FCN network could significantly reduce computation and introduces invariance to small translation of input image [21]. However, 2-D spatial precision is lost during downsampling in pooling layers [30], resulting in an output of lower resolution. A typical solution is to upsample the output using a learned deconvolution, as shown in Fig 8. Nevertheless, this is a sub-optimal approach since the start point is the sub-sample function of the input image [15]. To compensate the resolution loss, we apply a skip architecture that combines the coarse layer with the shallow layer to refine the spatial precision of the output, as introduced by Shelhamer et al.[14].

**Fig 9:**
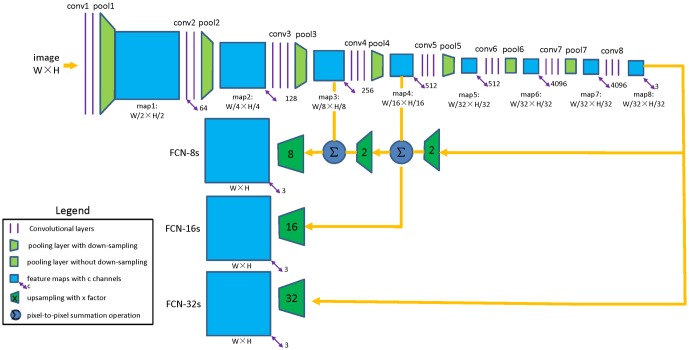
FCN-VGG16 network with skip architecture.

Fig 9 illustrates the basic architecture of FCN transfered from a pre-trained VGG 16-layer net. Like most of the applications of transfer learning, the last classifier layer of VGG-16 was discarded, and all the fully connected layers was transformed to convolutional layers. A 1**╳**1 convolutional layer *conv8* was appended and a feature map *map8* with dimension W/32 **╳** H/32 **╳** 3 was generated (3 channels of the feature map respond to 3 classes: *Rice*, *Weeds* and *Others* class, respectively). However, to generate a weed cover map, the class map should be the same size as the input image, so the last feature map *map8* was upsampled to full resolution using deconvolutional layer, as introduced by Shelhamer et al.[14]. The idea of skip architecture can be applied as followed: (1) if the last feature map *map8* is directly upsampled by a factor of 32, then a class map at full resolution is obtained, which is called FCN32s; (2) if the feature map *map4* is fused with 2 **╳** upsampling of feature map *map8* using the element-wise summation and the fusion result is upsampled by a factor of 16, the result is called FCN-16s; (3) if the feature map *map3* is fused with 2 **╳** upsampling of the prediction fused from *map4* and *map8* and the fusion result is upsampled by a factor of 8, then the output class map is called FCN-8s; (4) If this fashion is continued by combining even lower layers, a dense class map containing more detailed information will be obtained.

#### Evaluation metrics

In this work, four evaluation metrics were applied on classification accuracy, as introduced by Shelhamer et al.[14]. Let n_ij_ denote the number of pixels of class i and predicted as class j, t_i_ be the number of pixels belonging to class i, and c be the number of classes.

$$\text{pixel}\;\text{accuracy}:\frac{\sum_{i=1}^{c}n_{ii}}{\sum_{i=1}^{c}t_{i}}$$ (4)

$$\text{mean}\;\text{accuracy:}\frac{1}{c}\sum_{i=1}^{c}\frac{n_{ii}}{t_{i}}$$ (5)

$$\text{mean}\;\text{IU}:\frac{1}{c}\sum_{i=1}^{c}\frac{n_{ii}}{t_{i}+\sum_{j=1}^{c}n_{ji}-n_{ii}}$$ (6)

$$\text{frequency}\;\text{weighted}\;\text{IU}:{\left(\sum_{k=1}^{c}t_{k}\right)}^{-1}\sum_{i=1}^{c}\frac{t_{i}\times n_{ii}}{t_{i}+\sum_{j=1}^{c}n_{ji}-n_{ii}}$$ (7)

The proposed metrics covered both pixel accuracy and region intersection over union (IU), which will systematically evaluate the performance of the tested algorithm. According to Gabriela.et al. [31], IU was an object-based overlap measure typically used for imbalanced datasets. Since the pixels of *Weeds* class was in minority in our dataset, so the mean IU was the main metrics for our experiments.

In the following experiments, all the models were trained on the training dataset (892 imagery, as shown in the section “Materials and method”), and evaluated on the validation dataset which is independent from the training data (200 imagery in total). All the experimental results reported were based on the validation dataset.

## Results and discussion

### Experiments with transfer learning

Since our training dataset is relatively small, the training images are highly correlated, which may cause overfitting during training process [15]. Instead of training from random initialization, the pre-trained CNNs was applied for fine-tuning to accelerate the training process and avoid the problem of overfitting. The AlexNet, VGGnet [28] and GoogLeNet [29] were cast into FCNs and the output was directly restored to full resolution with in-network upsampling, as shown in Fig 8.

Table 1 compares the performance of FCNs based on different pre-trained CNN architectures, using different metrics covering the accuracy and efficiency. Though Shelhamer et al.[14] report an accuracy decrease of GoogLeNet in [14], our implementation of GoogLeNet used a pre-trained GoogLeNet for fine-tuning and achieved a performance close to VGG 16-layer net. In contrast with AlexNet and GoogLeNet, the FCN network based on the pre-trained VGG-16 achieved the highest score in terms of prediction accuracy, so the VGG 16-layer net was chosen as the basic architecture extended to FCN.

**Table 1:** Comparison of FCN with different pre-trained CNNs. The inference time is the average number of 200 trials for 1000 *1000 images using a GTX 1060.

Evaluation metrics	FCN-AlexNet	FCN-VGG16	FCN-GoogLeNet
***pixel acc*.**	*0*.*862*	*0*.*923*	*0*.*919*
**mean acc.**	0.724	0.795	0.776
**mean IU**	0.649	0.728	0.713
**f.w. IU**	0.807	0.876	0.869
**inference time**	0.106 s	0.318 s	0.169 s

The experimental results proved that all the transferred networks achieved a remarkable performance, even the worst model achieved approximately 89% of the state-of-art performance, as shown in Table 1. The VGG-16 based network achieved the highest classification accuracy, with the mean IU up to 0.728. Nevertheless, the Alex-Net based network outperformed other models in terms of efficiency, with the average speed of 0.106 second per image, demonstrating that this model could be considered in the application asking for real-time processing.

**Fig 10:**
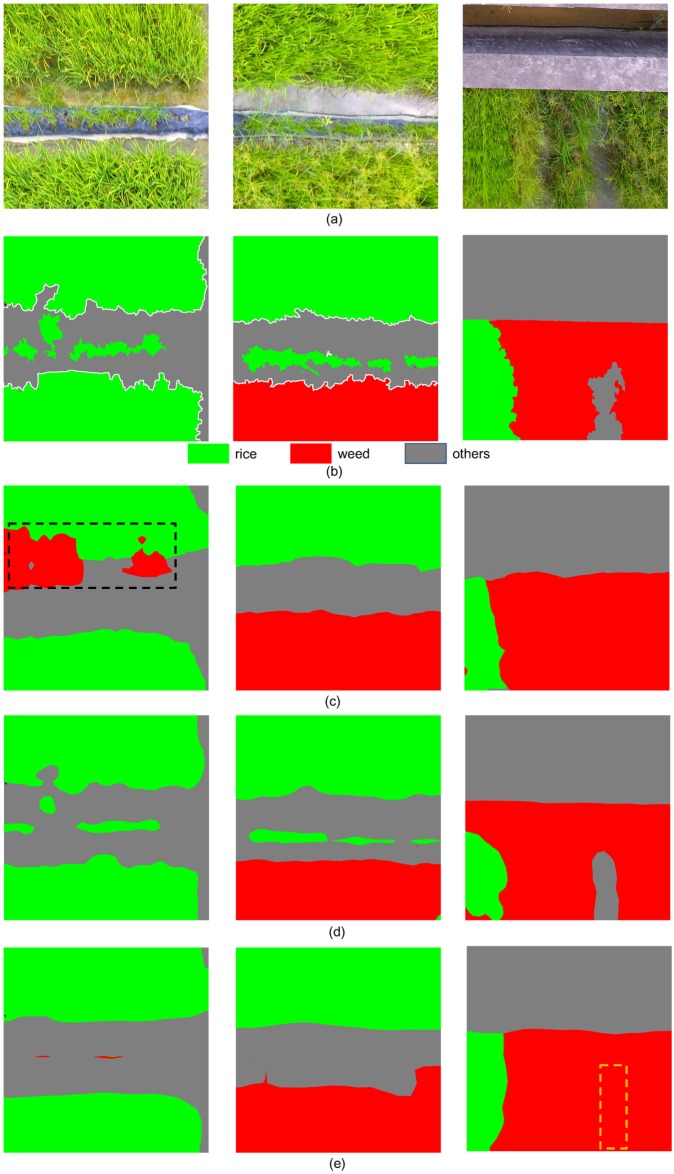
Classification results of FCN with different pre-trained CNNs. (a) UAV imagery; (b) corresponding GT labels; (c-e) Results obtained by FCN-AlexNet, FCN-VGG16 and FCN-GoogLeNet, respectively.

Fig 10 listed the results of FCNs based on different pre-trained CNNs. From Fig 10, it can be observed that FCN-AlexNet misclassified the *Rice* as *Weeds* (with black dashed boundary), and FCN-GoogLeNet misclassified *Others* as weeds (with yellow dashed boundary), while FCN-VGG16 correctly distinguished different classes.

### Experiments with skip architectures

To make up the resolution loss caused by downsampling operations of the network, the skip architecture was applied to improve the prediction accuracy. In this work, FCN-32s, FCN-16s and FCN-8s were taken for the experiments of skip architectures, as Fig 9.

Table 2 lists the performance of FCN network with different skip architectures. First, the coarse output of VGG-16 net was directly upsampled to full resolution (FCN-32s), resulting in a mean IU of 0.728. Following the idea of skip architecture, the lower layer *pool4* was fused with the coarse output before upsampling (FCN-16s). With this architecture, the mean IU was raised by 0.021 to 0.749, which means that the fusing strategy could actually improve the classification accuracy. This fashion was continued and even lower layer *pool3* was fused to the network (FCN-8s), achieving a minor improvement to 0.752 mean IU. At this time, the FCN network has met diminishing returns, both in terms of mean IU and other metrics, so we stop fusing even lower layers.

**Table 2:** Comparison of the of FCN network with different skip architectures, based on the pre-trained VGG 16-layer net. Learning is end-to-end, pixel-to-pixel.

Evaluation metrics	FCN-32s	FCN-16s	FCN-8s
***pixel acc*.**	0.923	0.928	0.935
**mean acc.**	0.795	0.811	0.807
**mean IU**	0.728	0.749	0.752
**f.w. IU**	0.876	0.886	0.892
**inference time**	0.318s	0.317s	0.323s

**Fig 11:**
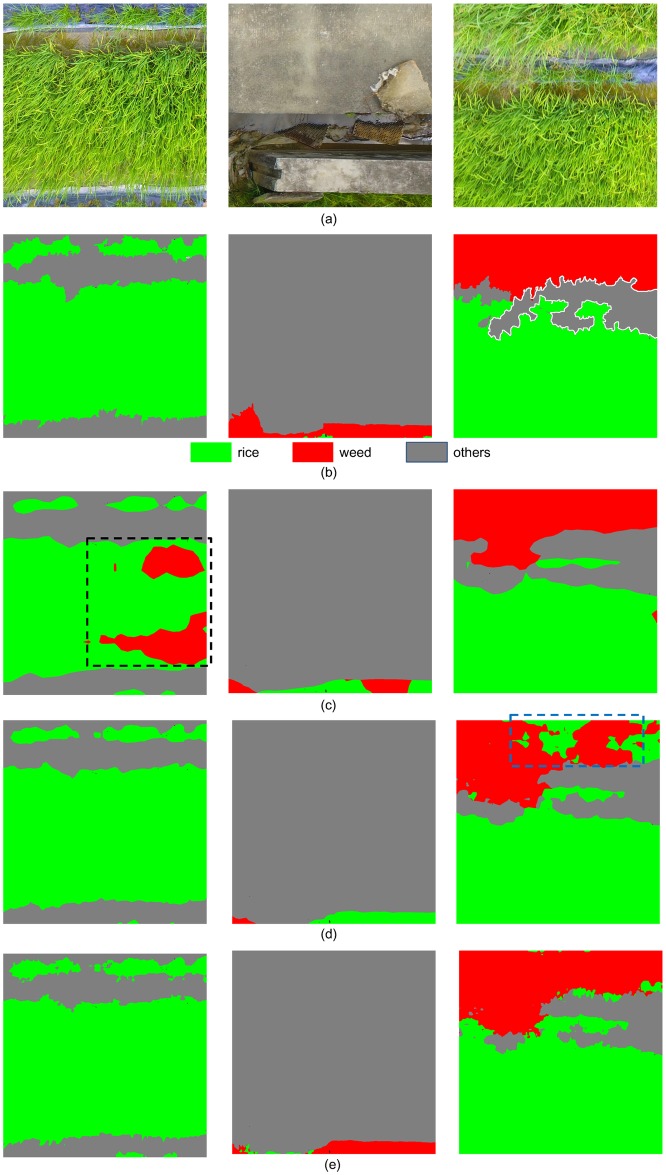
Classification results of FCN network with different skip architectures. (a) UAV imagery; (b) corresponding GT labels; (c-e) Results obtained by FCN-32s, FCN-16s and FCN-8s, respectively.

Fig 11 lists the classification results of FCN-32s, FCN-16s, and FCN-8s. From Fig 11, it can be observed that FCN-32s misclassifies *Rice* as *Weeds* (in black dotted boundary), and FCN-16s misclassifies the *Weeds* as *Rice* (in blue dotted boundary), while FCN-8s achieves the highest accuracy in rice recognition and weed detection. Also, the border of FCN-8s output is more closer to the GT labels, compared with other architectures.

### Comparison with other methods

#### Patch-based CNN

**Fig 12:**
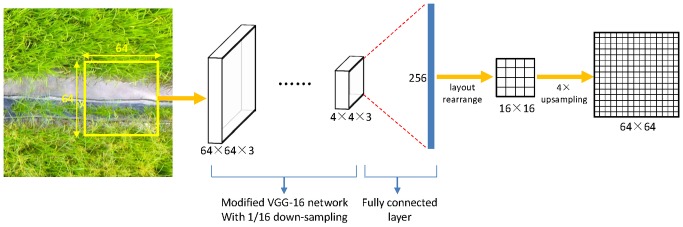
The network architecture of Patch-based CNN.

The network architecture of Patch-based CNN is shown in Fig 12. Different from the architecture used in [13, 32], the modified pre-trained VGG-16 network with 1/16 down-sampling factor was applied as the feature extractor. The stride of the last pooling layer was set to 1, because fusing the previous feature map *map4* with the coarse output could significantly improve the classification accuracy, as shown in Table 2. Following the idea of Mnih V [32], a fully connected layer was appended to the last pooling layer of VGG-16 network, mapping the previous layer to 256 neurons, which is later rearranged into a 16×16 prediction areas. Finally, an upsampling operation was performed by a factor of 4 to restore the resolution.

#### Pixel-based CNN

Compared with FCNs and Patch-based CNN, the method of Pixel-based CNN is a classification method in an “image-label” mode, which will classify a specific patch into a single label. Following the idea of Sharma et al.[33], classification for the imagery was processed pixel by pixel, considering the neighboring patch centered at the processed pixel. Different from the architecture used in [33], the pre-trained AlexNet was applied for fine-tuning. The reason for this choice is that our data is high-resolution imagery, which is close to the images in ImageNet database, so directly applying the model pre-trained on ImageNet could significantly speed up the convergence and improve the performance.

#### Experiments and comparison

In the experiment on Patch-based CNN, the patch size was set to 200, so the 200×200 patches are imported to the classifier. In the experiment on Pixel-based CNN, the performance of 5×5, 25×25 and 51×51 neighboring was compared, and the optimal value 25×25 was selected as our experimental parameter. Table 3 lists the comparison of performance of Patch-based CNN, Pixel-based CNN and FCN-8s on our validation dataset. It could be seen from Table 3 that FCN approach outperformed other methods in terms of accuracy and efficiency.

**Table 3:** Performance comparison of Patch-based CNN, Pixel-based CNN and FCN-8s.

Evaluation metrics	Patch-based CNN	Pixel-based CNN	FCN-8s
*pixel acc*.	0.727	0.651	0.935
mean acc.	0.611	0.608	0.807
mean IU	0.528	0.446	0.752
f.w. IU	0.651	0.551	0.892
inference time	0.338s	721.776s	0.323s

Table 4 lists the confusion matrix of three approaches. From Table 4, it is obvious that FCN network achieved the highest classification accuracy for all classes. Especially for the classification of *Weeds*, the accuracy of FCN network was up to 0.883, which is significantly higher than other approaches (Patch-based CNN 0.005, and Pixel-based CNN 0.455).

**Table 4:** Confusion matrix of three approaches for the validation dataset.

Algorithm	GT/ Predicted class	Rice	Weed	Others
Patch-based CNN	**Rice**	**0.971**	0.005	0.024
	**Weed**	0.953	**0.005**	0.042
	**Others**	0.154	0.016	**0.830**
Pixel-based CNN	**Rice**	**0.445**	0.261	0.295
	**Weed**	0.122	**0.455**	0.423
	**Others**	0.006	0.020	**0.974**
FCN-8s	**Rice**	**0.956**	0.017	0.027
	**Weed**	0.054	**0.883**	0.063
	**Others**	0.050	0.010	**0.940**

**Fig 13:**
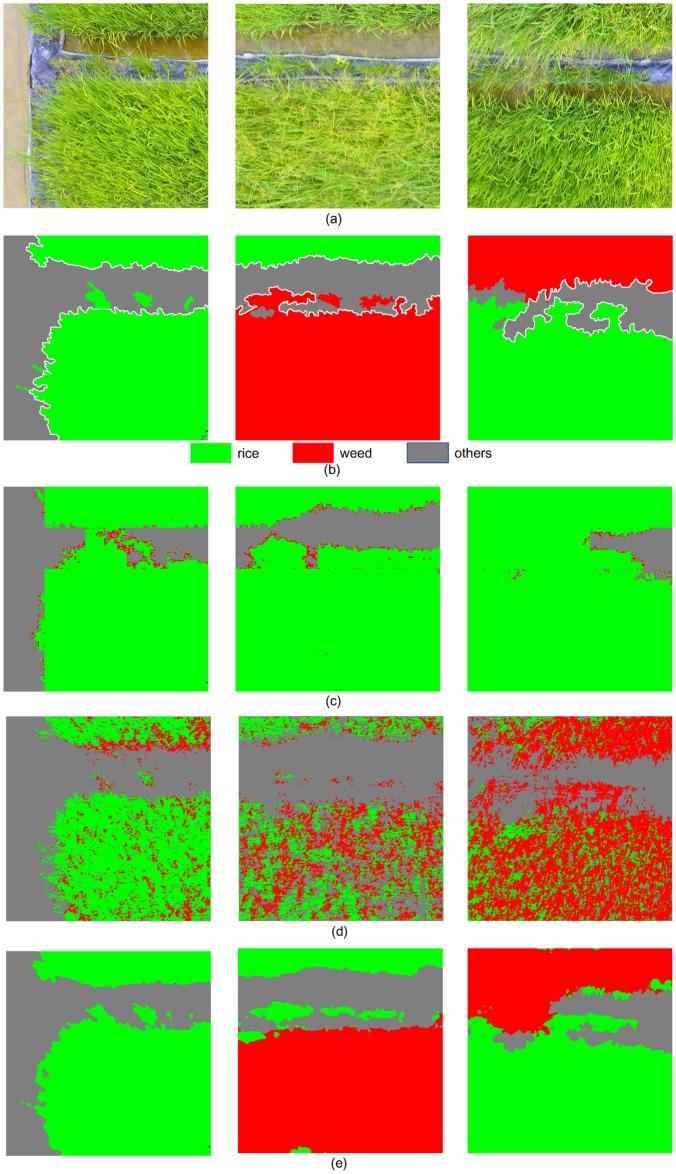
Classification results of three algorithms in comparison. (a) UAV imagery; (b) corresponding GT labels; (c-e) Results obtained by Patch-based CNN, Pixel-based CNN and FCN-8s, respectively.

Fig 13 lists the classification results of three algorithms in comparison. From Fig 13, it is obvious that the weed cover map generated by FCN network is more precise than other approaches.

In the Patch-based CNN algorithm, each patch is input to the network as an image, and the context within the patch is taken into consideration. However, the relationship between different patches is ignored, result in the discontinuities between patches. In comparison, the FCN method takes the whole image as input, which will consider the whole image overall and seamlessly. According to results of Patch-based CNN, most of the *Weeds* were misclassified as *Rice*, as Fig 13(c). From Table 4, the proportion of *Weeds* pixels classified as *Rice* was up to 0.953, which means that almost all the *Weeds* are recognized as *Rice*. One possible reason for this misclassification is that, the weeds are much less than rice in the training dataset, so the network was trained with rice data for much more iterations. Another possible reason is that weeds are similar with rice, both in color and texture features, so the network is more likely to classify the *Weeds* as *Rice*. Compared with Patch-based CNN, the FCN method uses patch-wise training strategy and can mitigate the problem of data imbalance, so the weeds in the imagery can be correctly classified, as shown in Fig 13(d).

In the Pixel-based CNN algorithm, the whole image is processed pixel by pixel. For the classification of each pixel, its surrounding 25×25 neighborhood is input to the AlexNet. However, the classification accuracy of Pixel-based CNN is much lower than FCN (Table 3), and change of the neighborhood size made no noticeable improvement. To speeding the computation, we set the batch size to 1000, which meant that the network will handle 1000 samples at one iteration, while the inference speed was still slower than FCN since too much redundant computation were preformed over the the neighborhoods. Compared with Pixel-based CNN approach, the FCN method took the whole image as input, resulting in better performance both in terms of accuracy and efficiency. As shown in Fig 13, a lot of *Rice* was misclassified as *Weeds* with Pixel-based CNN, while FCN generated a more accurate class map.

It can be seen from the comparison results that FCN method outperformed other approaches in terms of accuracy and efficiency. Also, the research on FCN and weed mapping could be expanded to operational situations of SSWM. Usually, the weed cover map produced by FCN method can be converted to a prescription herbicide treatment map [3] and then be transferred to machinery embedded with variable-rate spraying system. Variable-rate spraying system uses the prescription map to change flow rates according to weed densities of different sites, which may significantly promote the herbicide saving while enhancing the herbicide effectiveness.

## Conclusions

In this paper, UAV imagery was collected over a rice field, and then the FCN method was proposed for semantic labeling and weed mapping of the imagery. Transfer learning was used to improve the generalization capability and skip architecture was applied to increase the prediction accuracy. After that, the FCN network was compared with another two deep learning approaches. Experimental results demonstrated that our FCN network outperformed others, both in terms of accuracy and efficiency. Especially for the recognition of weeds, our FCN approach achieved the highest accuracy compared with other methods.

However, FCN is a fully supervised algorithm, and the training and updating of the network rely on large amount of labeled images, which requires extensive manual labeling work. This requirement limits the application of this method. So in the future of this work, we plan to introduce the weak-supervised learning and unsupervised learning algorithm to reduce the manual labeling work and enhance the ease of application.
